# Medical Fads and Faddists

**Published:** 1895-03

**Authors:** Le Roy Dibble

**Affiliations:** Kansas City, Mo.


For Texas Medical Journal.

### MEDICAL FADS AND FADDISTS.

BY LE ROY DIBBLE, M. D., KANSAS CITY, MO.

[Read before the Jackson County (Mo.) Medical Society.]

THE rise and fall of medical fads in the world's history would form a curious medley, if accurately written. In discussing them, I shall confine myself to the most prominent that have arisen in the last quarter of a century,—those that have come under my own observation.

There seems to be an innate tendency in the minds of certain individuals to run after fads. This mania is not confined to medical men, by any means. Unhappily, the medical man, like the theologian, deals in a kind of hidden mystery, largely founded on faith. It is not so long ago, in fact, that medical science, so-called, was made up largely of charms and superstitious incantations, as witness the savage tribes of our own day and generation.

The author of this article, not many years ago, was in the Austrian Tyrol, and while wandering through a remote village, was surprised at a barber's sign. Three little brass dishes (our modern pus pan) attracted my observation, and hanging over them was a white horse's tail. Entering into conversation with the proprietor, I learned that he was not only the village barber, but the village surgeon as well. He shaved the heads of his customers, and also bled them in the spring and fall. I had heard of these things, but had never seen them. My curiosity being excited, I asked him about his business, and was informed that he shaved or bled his customers for ten kreutzers. I asked him the use of the white horse's tail, and was informed its function was to be waved over the dish while the blood was flowing, to prevent the evil spirits from entering the open vein. The barber's pole, as seen in this country, is never seen there, and few people know the significance of the alternate red, blue and white stripes. The red denotes arterial blood, the blue, venous, and the white stripe the bandage to be applied after the operation. So, you see, that even in a highly civilized country, in these remote mountain regions, such superstitions still remain. They only differ in kind to our more modern superstitions, that have been changed into fads. In my short career, I can look back over the wrecks of a startling number of such that have shot athwart the medical skies in meteoric showers, only to be extinguished in nothingness, after leaving an ugly scar on the hands of the party holding them when they exploded.

The first fad that I recollect was cundurango. It had the indorsement of one vice-president, the clergy (they nearly all indorse a nostrum if it is a gift), and many eminent laymen. It had a brief but inglorious career as a sure cure for cancer. Only the older members of the profession remember it.

The next was chloral hydrate. It was to relieve all the pains that flesh is heir to, and was vaunted to supercede the old and tried opium in all its forms. By trial, it has been relegated to the realm of "plain drunks," and not very useful even in these cases.

Then the laity had its turn at the liver-pad, and what a heaven given boon it was to be; how beautiful in theory—as theories usually are. All you had to do in the morning was to adjust your liver-pad, and aches and pains vanished as if by magic. It also "fell down," after the advertisers had emptied the livers of the public and filed their pockets. The copper wire worn around the waist mingling with the acid perspiration and excreta of the body was said to act as a perpetual battery and give you perfect "electropoise," whatever that means.

Soon after came the electric disk, worn around the neck (a la savage), price only 50 cents. It was so cheap it was said "if it didn't do any good it wouldn't do any harm," and yet it filled the vacuum of a "long felt want."

Then dear old General Pleasanton solved the problem of life by evolving that marvelous theory of blue glass, and the "dear people'' fairly reveled in the luxury of sunlight modified by blue tints.

Brown-Sequard's elixir next had its ''running.'' It was to turn back the ''tide of time,'' and make old men young again. The injected ''lean and slippered pantaloon with shrunk shanks,'' was to enter immediately into the dissipations, follies and excesses of callow youth. Fortunately for the human race, it did *not* succeed. It emanated in the senile brain of a once honored scientist. Peace to his ashes. Let us kindly draw the veil of charity over his weakness; we all, at some time in our lives, have done some things we would not care to have inscribed on our tombstone.

Koch's lymph, that was to destroy the fell destroyer of a large portion of the human race, next shot like a comet into the medical horizon. It was incubated in the brain of a much vaunted scientist. Its birth was heralded to the world under the sheltering wing of royalty. It proved to be a failure also; and after much coddling and nursing, it was abandoned and died.

Mythel violet, that mysterious nothing in color, that was to stay one of the most dreaded diseases that the human race is heir to, next was heralded as a great wonder. It vanished in a night as it were, and left naught behind but doubly disappointed hopes of those afflicted with cancer.

Christian science, emanating in the fertile brain of imagery in that land of ''steady habits, isms and beans,'' takes no note of material things—even life itself, or its tenets, is only a myth. But why dub it Christian more than Pagan, Mohamedan or Brahmin, the writer knows not (and there is surely no science about it), since all known religions are tinctured with the same mystery. It seems a kind of ''black art,'' as old as the human race, and those who revel in mystery, and shout about things they know the least about, will revive it, from time to time, under a different name, and modify it to suit new conditions. As a curative agency, it seems to be a little less tangible, if possible, than homeopathy.

Osteopathy and orificial surgery seem at present to be a local affliction (it may become general later). All disease, according to its ritual, is caused by dislocation of the bones. When ''set,'' the disease they have caused disappears. It don't mention a cure where wheels are loose in the head. They will be adjusted by some new fad yet to be discovered.

And now, ''finally, brethren,'' we come to the last, and therefore the greatest fad—antitoxine. I am aware that I am now treading on dangerous ground. So many enthusiasts are swearing by this new discovery. I hope they will not have occasion to *swear at it.* Let us look over the ground carefully and note results. How many of these cases that are reported as diphtheria, really are such? Without the proof of cultures, the evidence is absolutely worthless. Of thirty cases recently reported in Berlin, but five gave the cultures, and three out of the five died. I was somewhat startled when I read the label on the bottle: "Use your usual remedies;" and if you do, then what? Are we to suppose no cases of diphtheria have been cured by the "usual remedies?" This is "shot-gun" practice with a vengeance. Who knows which shot killed or cured? Let us take a dispassionate view of the field, hold fast to that that is proven good, and keep out of the public prints until the worth of a proposed remedy is thoroughly established, lest there be another rude shock in store for the medical profession. It can't stand many such as Koch's lymph gave us, and hold up its head in the scientific world. I hope we have found something; but let us wait and see.

I shall only mention a few of the more prominent fadists:

Dr. Fawning is a gentleman with an oleaginous, unctuous countenance, who washes his hands in invisible soap and water, and assures you with a profound bow that your last expression is his exact views better expressed. There is not a new fad but he is the first to embrace it and extoll its virtues, and by this means he get his name in the public prints so dear to his heart, and incidentally much *free advertising*. He is often a delegate to read a paper before some distant society, and heralds the fact through the daily press, but usually fails to appear. If he does, minus a paper, for he might be obliged to express some positive views on some subject.

Dr. P. D. Q.—Invariably an ignorant, blatant charlatan, a product of our defective system of medical education. Too much should not be expected, however, from the fountain-head from which he sprang. A fountlet is seldom more pure than its source. His real abilities lie in the direction of a pot-house politician, carrying a hod or trundling a wheelbarrow. He's a shining example of a medical "misfit."

Dr. Buncombe—A veritable "Bombastes Furioso, or army in buckram," a braggart, a bully, and a blusterer in the profession. If by any chance he can worm himself into your case, he will insinuate to the patient and friends that if you had done so and so, as he would have done, all would be well. He is a man to be avoided as you would a cyclone.

Dr. Mounte-Bank—A man who spreads himself before admiring constituents as ignorant as himself; a man who never learned the difference between being *noted* and *notorious*. He is usually from some remote region and assumes the proprietorship of a personal organ to advertise himself, before he has lost his shambling gait from driving cows to pasture, gotten the cockle burrs out of his hair and the odor of the barnyard from his clothing.

Dr. Q. A. Explorer—A gentleman who is always looking for a small piece of intestine that God in his munificence had left over when he created man. The knowledge of its existence stimulates the faddist to great exertions, in order to relieve his patient of this superfluity and incidentally of any surplus cash the victim may possess. Wealthy people have appendicitis that demands immediate operation, poorer ones have colic or plain belly-ache that is usually relieved by peppermint and sugar, or something equally simple and efficacious. In after years, when the faddist contemplates with wonder the marble shafts he has raised to an over wrought imagination, it is to be hoped he will drop a silent, but penitent tear over the "memoriams" that confront him.

Dr. W. B. Ripper (specialist), from New York or Philadelphia, "after attending a post-graduate course of six weeks, etc., etc., and now being *thoroughly qualified*," parades himself before every society that will listen to his self-laudation. He seldom fails to tell the general practitioner how ignorant he is, even if he has grown gray in practice, and he himself, only a beardless stripling. The knowledge that such an organ exists as an ovary, is like planting a red flag in the face of a mad bull. In after years it is hoped he will look back and sigh at the *barren waists* he has created.

Dr. Plainliar—A man of marvelous resourses (in lying), a veritable miracle worker, who reports "my last thousand cases of lapciotomy without a death." This marvelous record is usually exhibited before the Podunk Valley Mutual Admiration Society (limited) and veritably "knocks down the groundlings" who furnish the champagne and "funeral baked meats" after the performance.

Dr. Steadfast—A plain unassuming man, a scholar and a gentleman, with no fads and no superfluous appendages to his name.

He stands like a lighthouse or a storm swept shore. No matter how severe the tempest of fads and "isms" the light of his good common sense shines steadily and constantly. In the community where he dwells, he has the respect of the thinking educated people. He is never rich, his heart is too big, for no worthy person comes to him and is turned away. And when he is called to that "great beyond," he is sincerely mourned by rich and poor alike as a friend and counsellor, and his memory treasured in after years as the "noblest work of God, an honest man."

